# Reconstruction of Metabolic–Protein Interaction Integrated Network of *Eriocheir sinensis* and Analysis of Ecdysone Synthesis

**DOI:** 10.3390/genes15040410

**Published:** 2024-03-26

**Authors:** Tong Hao, Zhentao Song, Mingzhi Zhang, Lingrui Zhang, Jiarui Yang, Jingjing Li, Jinsheng Sun

**Affiliations:** 1 Tianjin Key Laboratory of Animal and Plant Resistance, College of Life Sciences, Tianjin Normal University, Tianjin 300387, China; skyht@tjnu.edu.cn (T.H.); a953835912@gmail.com (Z.S.); 18404964001@163.com (M.Z.); ztt11235813@163.com (L.Z.); yanng0524@163.com (J.Y.); 2 Tianjin Fisheries Research Institute, Tianjin 300211, China; jingjingli206@163.com

**Keywords:** integrated network, metabolic network, protein–protein interaction network, *Eriocheir sinensis*, ecdysis

## Abstract

Integrated networks have become a new interest in genome-scale network research due to their ability to comprehensively reflect and analyze the molecular processes in cells. Currently, none of the integrated networks have been reported for higher organisms. *Eriocheir sinensis* is a typical aquatic animal that grows through ecdysis. Ecdysone has been identified to be a crucial regulator of ecdysis, but the influence factors and regulatory mechanisms of ecdysone synthesis in *E. sinensis* are still unclear. In this work, the genome-scale metabolic network and protein–protein interaction network of *E. sinensis* were integrated to reconstruct a metabolic–protein interaction integrated network (MPIN). The MPIN was used to analyze the influence factors of ecdysone synthesis through flux variation analysis. In total, 236 integrated reactions (IRs) were found to influence the ecdysone synthesis of which 16 IRs had a significant impact. These IRs constitute three ecdysone synthesis routes. It is found that there might be alternative pathways to obtain cholesterol for ecdysone synthesis in *E. sinensis* instead of absorbing it directly from the feeds. The MPIN reconstructed in this work is the first integrated network for higher organisms. The analysis based on the MPIN supplies important information for the mechanism analysis of ecdysone synthesis in *E. sinensis*.

## 1. Introduction

Cellular networks reveal and reproduce the network topology of interactions between biological elements to explore the complex biological mechanisms and functions based on the systematic view. With the in-depth study of various networks, researchers have recognized the limitations of a single type of network. Therefore, the integration of multiple types of networks has caused more interest [1]. The functions of various molecules in organisms (genes, proteins, metabolites, etc.) are expressed through the interactions between each other. It is necessary to make a holistic analysis of all relevant components through the integrated networks to comprehensively understand the complex physiological functions. Among these pieces of research, the study on the integrated networks involving genome-scale metabolic networks (GSMNs) is the most in-depth. In recent decades, the integration of metabolic and regulatory networks has become an important direction. In 2004, Plasson’s group [2] reconstructed the first integrated genome-scale computational model of a transcriptional regulatory and metabolic network *i*MC1010 through regulatory flux balance analysis (RFBA). This network contains 1010 *Escherichia coli* genes, including 104 regulatory genes, which regulate the expression of 479 genes out of 906 genes in the *E. coli* metabolic network. This network is not only able to predict the high-throughput growth phenotypes but also indicate the transcription factors that play an important role in regulating metabolic pathways and identify the previously unknown components and interactions in the metabolic and regulatory networks. Sriram and Nathan [3] proposed the probabilistic regulation of metabolism (PROM) method and reconstructed the metabolic-regulation integrated network of *E. coli* and *Mycobacterium tuberculosis*. Jiang et al. [4] reconstructed the metabolic-transcriptional integrated network of *Corynebacterium glutamicum* using the relationship graph method combined with the public database and literature database resources. This network contains 1384 reactions, 1276 metabolites, 88 regulators, and 999 transcriptional regulatory interactions. The relationship graph method integrates the metabolic network and transcriptional regulatory network taking the relationship between gene and enzyme as a bridge and forming the integrated network containing the relationships among regulators, target genes, enzymes, and reactions. The advantage of this integrated network is that it has discovered some two-level complex regulatory relationships between transcription and metabolism within cells, which cannot be obtained in a single metabolic network or transcriptional regulatory network. Jason et al. [5] proposed a TIGER toolbox, which can be used to integrate the genome-scale metabolic network, expression data, and transcriptional regulatory network. Sriram Chandrasekaran [6] proposed an integration method for the metabolic and regulatory networks based on the PROM (probabilistic regulation of metabolism) and GEMINI (gene expression and metabolism integrated for network inference) methods. PROM was first applied to automatically reconstruct the integrated metabolic transcriptional regulatory network, and the GEMINI approach was subsequently used to curate the integrated network using the transcriptomics and phenomics data. In recent years, studies on the integration of transcriptional regulatory networks and signal transduction networks have increased. Wang et al. [7] reconstructed an integrated network of *Saccharomyces cerevisiae* by integrating the transcriptional regulatory and signal transduction pathways, taking the transcriptional factor as the bridge. In this network, the signal transduction pathways are represented by protein–protein interactions (PPI). This network was used to explore the stress response mechanism of *S. cerevisiae*. 

Compared to the two-network integration, the integration of multi-networks is much more difficult. On the small-scale network integration, Markus et al. [8] proposed integrated flux balance analysis (iFBA) to construct a three-network integration model of metabolism, transcriptional regulation, and signal transduction in *E. coli*. Jason et al. [9] proposed an integrated dynamic flux balance analysis (idFBA) method to construct a *S. cerevisiae* model. However, these two methods cannot be used for large-scale network reconstruction due to parameter settings limitations. On large-scale network integration, Jonathan et al. [10] collected information from over 900 data sources such as reviews, books, and databases to reconstruct a *Mycoplasma genius* whole-cell model, which includes metabolic, signal transduction, and transcriptional regulation data. They found that the whole-cell model made more quantitative predictions than a single metabolic network [11]. Carrera et al. [12] reconstructed an integrated network of metabolism, transcriptional regulation, and signal transduction in *E. coli* by combining high-throughput transcriptomics and phenomics data. This model has demonstrated a stronger ability in phenotype prediction than simple metabolic networks. However, the perturbed genes obtained from the gene perturbation analysis of the network only cover 23% of the GO entries of *E. coli*. Macklin et al. [13] reconstructed the first *E. coli* whole-cell model described based on large amounts of publications and experiments, which included the central dogma, metabolism, and regulation processes of *E. coli*. The *E. coli* whole-cell modeling project [14] expanded the scope of this model by including missing gene and small molecule functionality as well as increasing the number of possible nutrient conditions for simulated growth. The model covered 43% of characterized genes. Ahn-Horst et al. [15] further expanded the *E. coli* whole-cell model by adding the dynamics of the global regulator guanosine tetraphosphate, along with the dynamics of amino acid biosynthesis and translation.

Currently, most integrated networks are built on microorganisms, especially model microorganisms. For higher organisms such as animals and plants, due to the complexity of their biological systems and the lack of transcriptional regulation and signal transduction data, integrated network reconstruction has not yet been completed. The protein–protein interaction network (PIN) contains a large amount of information such as signal transduction proteins, transcription factors, transcription factor binding proteins, methylation proteins, etc. [16]. Therefore, multiple network integration functions such as metabolism, transcriptional regulation, signal transduction, and even protein modification can be achieved if the metabolic network is integrated with the PIN. This integration method can provide a convenient way for the reconstruction of the integrated cellular networks for higher organisms.

*E*. *sinensis*, commonly known as river crab or Chinese mitten crab, belongs to the phylum Arthropoda. *E*. *sinensis* is an important economic source of fishery [17]. Molting is an important characteristic of the growth of *E. sinensis*. The life of *E. sinensis* undergoes 10–20 times of molting. However, about 15–30% of the cultured *E. sinensis* experience precocious puberty, which is related to rapid gonadal development and premature termination of molting in juvenile mitten crabs [18]. Studies on the molting mechanism have an important impact on improving precocious puberty and increasing the economic value of *E. sinensis*. Researchers have explored the molting mechanism of *E. sinensis* from various aspects such as gene analysis, endocrine analysis, and omics analysis. Currently, some substances and pathways related to molting have been identified, such as ecdysone, ecdysone synthesis pathway, mTOR pathway, etc. [19,20,21]. However, the complete mechanism of molting is still not clear. An important reason is the lack of tools for systematic analysis of metabolic and regulatory processes in *E. sinensis*.

Currently, the GSMN [22] and PIN [23] have been reconstructed for *E. sinensis*, reflecting its metabolic and regulation system, respectively. However, neither the flux balance analysis of GSMN nor the shortest path analysis of PIN can simulate the connection of the metabolic and regulation process of molting. Therefore, in this work, we tried to combine the two networks to reconstruct a genome-scale metabolic–protein interaction integrated network (MPIN) of *E. sinensis*. This network contains both metabolic and regulatory information of *E. sinensis*. It is the first MPIN for higher animals. The MPIN was used to simulate the synthesis of ecdysone, which is a key substance in the regulation of *E. sinensis* molting, and identify the reactions and proteins that play a crucial role in ecdysone synthesis to reveal its metabolic and regulatory mechanisms. The results of this work are of great significance for in-depth research on multi-network integration and further analysis of the molting mechanism in *E. sinensis*.

## 2. Methods

### 2.1. Transformation of the Metabolic Network to a Reaction Graph

The GSMN is usually a metabolite graph with metabolites as points and reactions as edges. The reconstruction of an integrated network first requires a transformation of the metabolic network into a reaction graph with reactions as points and common metabolites between reactions as edges. The conversion method of the reaction graph is referred to in our previously published paper [24]. The currency metabolites in the GSMN *i*crab4665 of *E. sinensis* reconstructed in our previous work [22] were first removed, and then the network was converted to a reaction graph using the common metabolites as a bridge to connect the reactions. For example, if the product of reaction 1 is the substrate for reaction 2, reaction 1 and reaction 2 can be connected with the direction from reaction 1 to reaction 2. The coefficient of the reaction is determined by the amount of metabolites present in the reaction. All the reactions in *i*crab4665 except biomass reaction were converted to a reaction graph. The transport and exchange reactions for the new nutrients detected in this work compared to *i*crab4665 were also added.

### 2.2. Addition of the Biomass Equation

The biomass of *i*crab4665 was constructed merely based on the substance components of hepatopancreas. To obtain a more comprehensive biomass composition, we reconstructed the biomass equation based on five tissues of *E. sinensis*: hepatopancreas, gill, muscle, thoracic ganglion, and eyestalk. The healthy juvenile crabs were obtained from Tianjin Xieyuan Aquaculture Co., Ltd. (Tianjin, China). The crabs were reared in a plastic incubator (70 cm × 40 cm × 50 cm) at 20 ± 1 °C under natural lighting conditions. Prior to tissue collection, the crabs were subjected to low-temperature anesthesia by placing them on an ice plate. Samples from five tissues of the crabs were swiftly collected and immediately submerged in liquid nitrogen for flash freezing. Subsequently, they were stored in a −80 °C refrigerator for preservation. The samples of these tissues were mixed, and three biological replicates were sent to the Beijing Institute of Nutritional Resources for cellular component detection with each sample weighing approximately 300 g. The contents of 2 sugars, 20 amino acids, 30 fatty acids, 10 trace elements, and 5 nucleotides were detected using the standard method specified in “GB28050-2011 national food safety standard general principles for nutrition labeling of prepackaged food” [25]. The biomass equation was constructed based on the detection results of the biomass composition. The equation contains carbohydrates, amino acids, fatty acids, nucleotides, and trace elements. Most of these substances can be found and numbered according to the KEGG database, except five fatty acids, including tridecanoic acid, margaric acid, cis-10-heptadecenoic acid, heneicosanoic acid, and tricosanoic acid. These five fatty acids were numbered M00001–M00005 for the purpose of subsequent simulations. The content of each substance was converted into the coefficient in the biomass equation according to the method described in the literature [22], thus constructing the biomass equation for the mixed tissues with the ID “B00001”.

The biomass equation was subsequently added to the integrated network. The entire GSMN was searched for the reactions containing the substances in the biomass equation, which exist as products in the reaction. Then the reaction was connected to the biomass equation and formed a reaction–biomass integrated reaction (R-B IR) in the network. The coefficients of the substance in the biomass equation were assigned to the metabolic reactions, and the coefficients of the substance in the metabolic reaction were assigned to the biomass equation. For example, the equation of metabolic reaction R00010 is C01083[c] + C00001[c] --> 2 C00267[c], and the coefficient of glucose (C00267) in the biomass equation is 0.0942, so the R-B IR composed of R00010 and biomass equation is 0.0942 R00010[c] --> 2 B00001[c]. The R-B IRs are all irreversible. As some of the substances in the biomass equation cannot be synthesized through the network, the transport and exchange reactions of these substances were added and linked with the biomass reaction. These substances might be absorbed from the feeds. 

### 2.3. Reconstruction of Integrated Network

The GSMN *i*crab4665 and PIN for *E. sinensis* reconstructed in our previous study were integrated to reconstruct the MPIN of *E. sinensis*. Both *i*crab4665 and the PIN of *E. sinensis* contain information on unigene related to metabolic reactions or proteins. Therefore, unigene was used as a bridge to link proteins and reactions. The reactions in the GSMN and proteins in the PIN with the same unigene were linked to integrate the two networks, and each link was considered an IR of the MPIN. Considering the different components in an IR, there are 8 different types of IRs in the integrated network: the IR consisting of reaction–reaction linkage (R-R IR), the IR consisting of protein–protein linkage (P-P IR), the IR consisting of protein–reaction linkage (P-R IR), the IR consisting of metabolic and biomass reaction (R-B IR), the IR consisting of metabolic and transport reaction (T-R IR), the IR consisting of transport and biomass reaction (T-B IR), the IR consisting of transport and transport reaction (T-T IR), and the IR consisting of transport and exchange reaction (T-E IR). Among them, P-P IRs, P-R IRs, T-R IR, and T-T IR are reversible. R-B IR and T-B IR are irreversible. The reversibility of R-R IR was determined according to the relationship of reactions in the GSMN. The reversibility of T-E IR was determined according to the source of nutrients.

The flux constraints of reactions in the MPIN are crucial for simulations. The fluxes of the P-R IRs, T-R IRs, and T-T IRs were limited to (−1000, 1000) mmolg_DW_^−1^h^−1^. The flux constraints of the P-P IRs were set to be (−1, 1) mmolg_DW_^−1^h^−1^. This is because the interactions in the PIN are un-directional and the size of the PIN is much larger than that of the GSMN. There might be many pathways in the network consisting of proteins and reactions, for example, from reaction 1 to protein A, protein A to protein B, protein B to reaction 2, and reaction 2 to other reactions in the integrated network. If the fluxes of the P-P IRs were set too large, there would be a large amount of substances synthesized through proteins rather than metabolic reactions, which is obviously unreasonable. Therefore, reducing the weight of PIN by constraining the flux of the P-P IR to be a smaller value will decrease the influence of P-P IRs on the flux distribution and help to increase the accuracy of the simulation result. The fluxes of R-R IR, R-B IR, and T-B IR were directly determined according to their reversibility, with reversible IRs as (−1000, 1000) and irreversible IRs as (0, 1000) mmolg_DW_^−1^h^−1^. The flux of T-E IRs for nutrients that were absorbed from the environment was set to be (−5, 1000) mmolg_DW_^−1^h^−1^, and that for the nutrients synthesized by the network was set to be (0, 1000) mmolg_DW_^−1^h^−1^.

### 2.4. Analysis of the Ecdysone Synthesis Pathway

#### 2.4.1. Addition of the Ecdysone Synthesis Pathway

Ecdysone is an important substance involved in the regulation of molting in *E. sinensis* [26], but the ecdysone synthesis pathway in the MPIN is incomplete. Therefore, the missing reactions in the ecdysone synthesis pathway obtained from map00981 in the KEGG database [27] were added to the MPIN, enabling the network to synthesize ecdysone. Cholesterol is the precursor for the synthesis of ecdysone, which is commonly considered to be absorbed from feed. Therefore, the cholesterol T-T and T-E IRs were added for cholesterol transport with the flux constraints set as (−5, 1000) mmolg_DW_^−1^h^−1^.

#### 2.4.2. Determination of Biomass Synthesis Rates

The rate of biomass synthesis was determined according to the literature [28]. The specific growth rate of three different families of *E. sinensis* in indoor culture has a maximum value of 1.454%/day, which is 0.056 converting to the unit gDWd^−1^. Assuming that the supply of feeds is measured in days, the rate of biomass synthesis is fixed at 0.056, i.e., the upper and lower flux bounds of the biomass exchange reaction were both set to 0.056. The detailed process of unit conversion has been explained in *i*crab4665 [22].

#### 2.4.3. Identification of Metabolic Reactions and Proteins That Affect the Ecdysone Synthesis

Flux variation analysis was used to identify the IRs that affect the ecdysone synthesis. The ecdysone synthesis process was analyzed as follows: the flux distributions of the network and the maximum synthesis rate of ecdysone were calculated with the FBA algorithm taking the exchange reaction of ecdysone as the target function. Subsequently, the upper bound of the ecdysone exchange reaction was set to a smaller value of 1 mmolg_DW_^−1^h^−1^, and the calculations were performed again. The reactions with flux variations in the two times of simulations were identified as reactions affecting ecdysone synthesis. The metabolic reactions and proteins involved in these IRs were then collected. All simulations were performed with Matlab’s COBRA 3.0 toolkit [29]. For reactions affecting ecdysone synthesis, pathway and subsystem analyses were performed using the information obtained from the KEGG database. For proteins affecting ecdysone synthesis, the DAVID database (https://david.ncifcrf.gov/, accessed on 22 May 2023) [30] was used for GO function annotation. Since the *E. sinensis* PIN was reconstructed based on six model organisms, namely, *Homes sapiens*, *Drosophila melanogaster*, *Caenorhabditis elegans*, *Rattus norvegicus*, *Mus musculus*, and *S. cerevisiae*, when using the DAVID database for GO annotation, the species source was selected as the above six model organisms in turn until all the proteins were annotated. Three types of GO annotations were searched and analyzed: biological processes, molecular functions, and cellular components.

### 2.5. Analysis of Key Metabolic Reactions and Proteins Affecting Ecdysone Synthesis

In order to determine the key metabolic reactions and proteins that affect ecdysone synthesis in the IRs with flux variations in Section 2.4.3, two times of simulations were further performed for each IR. Firstly, the flux of an IR was fixed at its maximum value, which is its flux value at the maximum ecdysone synthesis rate, and the synthesis rate of ecdysone was calculated with the FBA algorithm. Subsequently, the flux of this reaction was fixed at its minimum flux value, which is its flux value at the ecdysone synthesis rate of 1 mmolg_DW_^−1^h^−1^, and the synthesis rate of ecdysone was calculated again. The synthesis rates of ecdysone in the two times of simulations were compared. If the change of rate is larger than 0.1 mmolg_DW_^−1^h^−1^, the IR was identified as a key reaction. The metabolic reactions and the proteins contained in this IR were considered the key metabolic reactions and proteins affecting ecdysone synthesis. 

## 3. Results and Discussions

### 3.1. The Reconstruction of MPIN

#### 3.1.1. Transformation of Metabolic Networks and Addition of Biomass Equations

The *i*crab4665 is comprised of 4665 unigenes, 2060 reactions, and 1891 metabolites [22]. There are 1897 metabolic reactions in *i*crab4665. These metabolic reactions were converted into a reaction graph. The graph contains a total of 1793 reactions, of which 405 are reversible reactions and 1388 are irreversible reactions. These 1793 reactions are connected by 971 common metabolites, with a total of 4318 edges connecting the reactions, in which 756 undirected edges represent reversible R-R relationships and 3562 directed edges represent irreversible R-R relationships. 

*i*crab4665 includes transport and exchange reactions for 81 types of nutrients required by *E. sinensis*. In this work, the biomass composition was detected based on five mixed organs, and the contents of water, 2 sugars, 20 amino acids, 30 fatty acids, 10 trace elements, and 5 nucleotides were obtained. Compared with *i*crab4665, this detection result obtained 8 more fatty acids that needed to be absorbed from the feeds. Therefore, their corresponding transport and exchange reactions were added to the network, increasing the number of nutrient transport and exchange reactions from 81 to 89. The transport and exchange reactions for the nutrients were all linked to the metabolic reactions and added to the reaction graph. The scale of GSMN as a reaction graph is shown in Table 1. 

#### 3.1.2. Preliminary Reconstruction of Integrated Network

The PIN of *E. sinensis* includes 8225 proteins and 148,524 interactions, corresponding to 31,507 unigenes [23]. The GSMN *i*crab4665 contains 4665 unigenes [22]. There are a total of 1723 common unigenes in the GSMN and PIN. Using the common unigenes as a bridge, reactions and proteins with the common unigene in the two networks are connected to integrate the two networks. The integrated network has a total of 10,301 nodes and 153,228 edges. Among them, 835 edges connect proteins and metabolic reactions, with 207 common proteins in the two networks. The scale of the preliminary reconstructed MPIN is shown in Table 2.

#### 3.1.3. Addition of Biomass Equation

The construction of the biomass equation is based on the content of each component in the biomass composition detection results of *E. sinensis* (Supplementary File S1). A total of 128 metabolic reactions were found connecting to biomass equations in the integrated network to form reaction–biomass IR (R-B IR), referring to 51 common metabolites connecting biomass equations and metabolic reactions. In addition to metabolic reactions, transport reactions can also be connected to biomass equations. However, due to the fact that some metabolites in the biomass equation can be generated through metabolic reactions, reconnecting the transport reaction of these metabolites with the biomass equation will result in the direct absorption of these metabolites from the feeds rather than synthesizing them through metabolic pathways. Therefore, the transport reactions of these metabolites cannot be connected with the biomass equation. Only the transport reaction of the metabolites that cannot be synthesized from metabolic reactions can be connected to the biomass equation. Finally, a total of 20 transport reactions were connected to the biomass equation to form T-B IR, with common metabolites including C02679, M0001, C08322, C16537, C08362, M00002, M00003, C01712, M00004, M00005, C06262, C00076, C00070, C00238, C00305, C00034, C01330, C00038, C01529, and C00020. The T-B IRs are irreversible. The T-T and T-E IRs of the biomass equation were also added for simulation purposes. The newly added nodes and interactions between the biomass equation and other reactions are shown in Table 3.

#### 3.1.4. Correction of the Model

**Figure 1:**
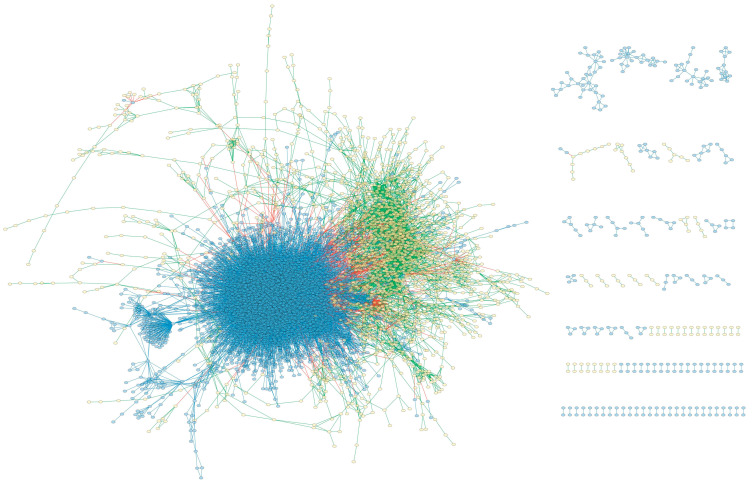
The MPIN of *E. sinensis.* Yellow nodes represent reactions; blue nodes represent proteins; R-R interactions are represented by green lines; R-P interactions are represented by red lines; and P-P interactions are represented by blue lines.

Compared to *i*crab4665, some new fatty acids have been added to the metabolic network part of the MPIN, but the metabolic pathways for these fatty acids are incomplete. Actually, the metabolic pathway of fatty acids in *i*crab4665 is also defective. The synthesis rate of biomass was 0 when the MPIN was used to simulate the biomass synthesis capability, which indicates the defects in the fatty acid metabolite pathways in the network. Therefore, the synthetic ability of all the fatty acids was verified. It was found that 11 fatty acids cannot be synthesized, including arachidic acid, cis-11-Eicosenoic acid, docosanoic acid, dihomo-γ-linolenic acid, erucic acid, cis-11,14,17-eicosatrienoic acid, 13c,16c-docosadienoic acid, lignoceric acid, EPA, nervonic acid, and DHA. After carefully checking the fatty acids metabolic pathways, the unsaturated fatty acid pathways were found to be highly incomplete. In order to complete these pathways, 23 reactions in the unsaturated fat acid pathway were added, including R12170, R12171, R12205, L00001, L00002, L00003, L00004, L00005, L00006, L00007, L00008, L00009, L00010, L00011, L00012, R08184, R08185, R08186, R08187, R08188, R08190, R08192, and R08273. Furthermore, T-R IRs, T-T IRs, and T-E IRs of these reactions were added for simulation needs. The synthesis capability of these unsaturated fatty acids was checked again after supplementing the fatty acid pathways. The reaction flux of linoleoyl-CoA, errocytochrome b5, reduced acceptor, and hydrogen sulfide remained at 0 mmolg_DW_^−1^h^−1^, indicating that the integrated network cannot synthesize these substances through the currently known knowledge. Therefore, the flux constraints of the T-E IRs for these four substances were set to be (−5, 1000) mmolg_DW_^−1^h^−1^, indicating that these substances are available from the environment. The flux constraints of the other 20 newly added T-E IRs were set to be (0, 1000) mmolg_DW_^−1^h^−1^, indicating that these substances are synthesized by the network and not available externally. In addition, since oxygen is essential for an organism’s metabolism, the exchange and transport reactions of oxygen were also added, and their flux constraints were set to be (−1000, 1000) mmolg_DW_^−1^h^−1^, which means that the integrated network can obtain oxygen from the outside environment. The final scale of the MPIN of *E. sinensis* is shown in Table 4 and Figure 1.

### 3.2. Analysis of the Ecdysteroid Pathway

#### 3.2.1. Addition of the Ecdysone Synthesis Pathway

The ecdysone synthesis pathway starts from cholesterol and can be mainly divided into two stages. The first stage is the formation of 7-dehydrocholesterol (7DC) from cholesterol catalyzed by the Neverland protein (NVD), and the 7DC subsequently generates 5β-diketol in the mitochondria. The second stage is the synthesis of ecdysone from 5β-diketol catalyzed by CYP306A1, CYP302A1, and CYP315A1. Finally, 20-hydroxyecdysterone is produced through the catalysis of CYP314A1. 

In order to assign MPIN the capability of ecdysis synthesis, 11 reactions in the ecdysone synthesis pathway have been added to the network, including R08132, R08136, R08139, R08140, R08137, R08133, R08135, R02373, R08141, R02374, and R08142. The added reactions were linked to other reactions and proteins in the MPIN to enable the network to synthesize ecdysone. Finally, 23 R-R IRs, 5 T-R IRs, and 6 P-R IRs were added, with their flux constraints set to (−1000, 1000) mmolg_DW_^−1^h^−1^. Furthermore, 1 T-T IR and 1 T-E IR for cholesterol transport were added. The scale of the MPIN after adding the ecdysone synthesis pathway is shown in Table 5 (Supplementary File S2).

#### 3.2.2. IRs Affecting the Synthesis of Ecdysone

The IRs that affect the ecdysone synthesis were analyzed with flux variation analysis. The flux of the biomass equation was first fixed to the experimental value, and the T-E IR of ecdysone was set to be the objective function. In the first simulation, the maximum rate of ecdysone synthesis was calculated with FBA, and the flux of each IR under this condition was recorded. The obtained maximum rate was 5.0 mmolg_DW_^−1^h^−1^. In the second simulation, the upper and lower bounds of the T-E IR for ecdysone were set to be a smaller value of (0, 1) mmolgDW^−1^h^−1^, which constrained the maximum synthesis rate of ecdysone as 1 mmolg_DW_^−1^h^−1^. The fluxes of the IRs in the MPIN under this condition were calculated and recorded.

**Figure 2:**
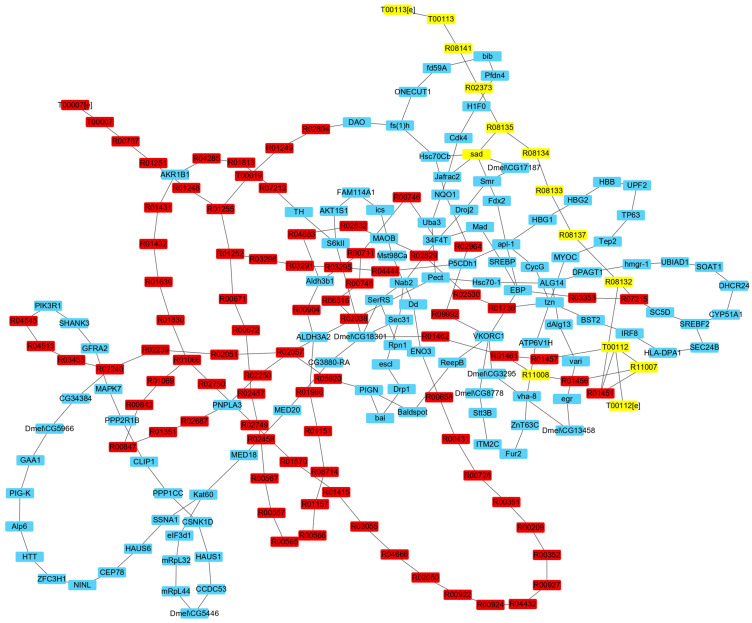
The network comprised of the IRs affecting ecdysone synthesis. Yellow nodes represent reactions in ecdysone synthesis pathway, red nodes represent other reactions, and blue nodes represent proteins.

Comparing the fluxes of the IRs in the two times of simulations, there were 489 IRs with flux variations. To avoid false positive results due to calculation errors, only the reactions with flux changes larger than 0.01 mmolg_DW_^−1^h^−1^ or smaller than −0.01 mmolg_DW_^−1^h^−1^ remained. Finally, 236 IRs were obtained after screening, including 27 P-R IRs, 116 P-P IRs, 81 R-R IRs, 6 T-T IRs, 3 T-R IRs, and 3 T-E IRs (Supplementary File S3). Figure 2 shows the network comprised of the IRs that affect ecdysone synthesis.

**Figure 3:**
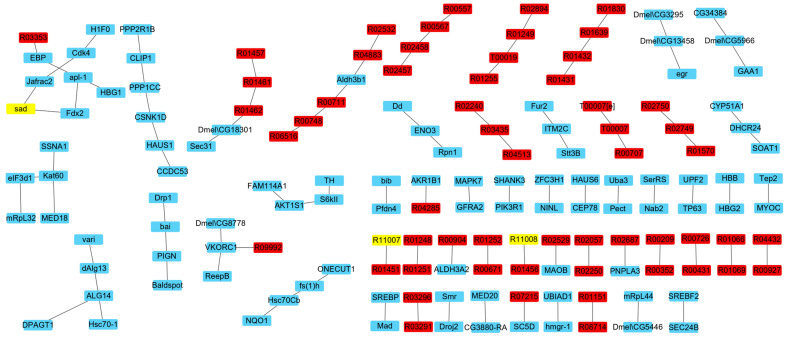
The network comprised of the IRs that compete with ecdysone synthesis. Yellow nodes represent metabolic reactions in the ecdysone synthesis pathway, red nodes represent other metabolic reactions, and blue nodes represent proteins.

**Figure 4:**
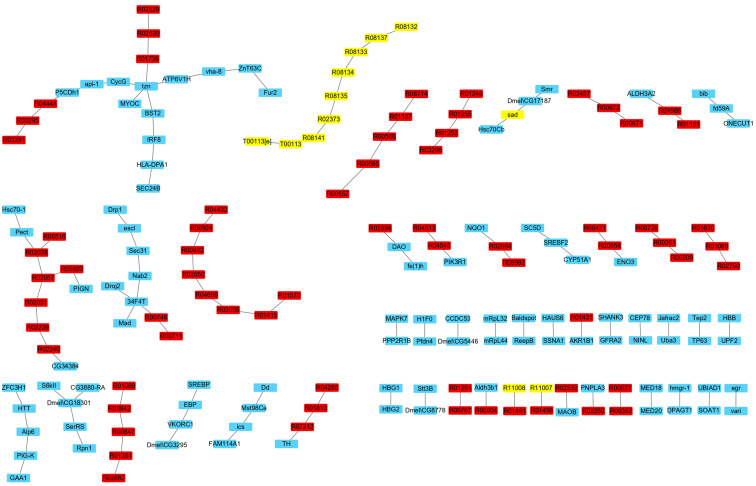
The network comprised of the IRs that promote ecdysone synthesis. Yellow nodes represent metabolic reactions in the ecdysone synthesis pathway, red nodes represent other metabolic reactions, and blue nodes represent proteins.

The IRs were further identified as IRs that compete with ecdysone synthesis, i.e., IRs with larger flux in the second simulation, and IRs that promote ecdysone synthesis, i.e., IRs with larger flux in the first simulation. The results showed that 105 IRs were competitive with the synthesis of ecdysone, including 58 P-P IRs, 8 P-R IRs, 32 R-R IRs, 2 T-T IRs, 2 T-R IRs, and 3 T-E IRs, in which three metabolic reactions in the ecdysone synthesis pathway were included. Figure 3 shows the network comprised of the IRs that compete with ecdysone synthesis. On the other hand, 131 IRs were found to promote the synthesis of ecdysone, including 58 P-P IRs, 15 P-R IRs, and 58 R-R IRs, in which 12 reactions in the ecdysone synthesis pathway were included. Figure 4 shows the network comprised of the IRs that promote ecdysone synthesis.

### 3.3. Analysis of Reactions and Proteins Affecting Ecdysone Synthesis

#### 3.3.1. Analysis of Reactions Affecting the Synthesis of Ecdysone

The IRs in the MPIN are actually linked pairs of reactions or proteins. The metabolic and transfer reactions (including transport and exchange reactions) contained in the IRs affecting ecdysone synthesis were extracted. A total of 97 reactions affecting the synthesis of ecdysone were extracted, of which 93 were metabolic reactions and the remaining 4 were reactions transporting nutrients, including glutamate, proline, cholesterol, and ecdysone. The reactions and proteins included in the IRs that compete with ecdysone synthesis were identified as competitive reactions and inhibitor proteins, respectively. The reactions and proteins contained in the IRs promoting ecdysone synthesis were identified as promotive reactions and promotive proteins, respectively. Finally, 62 competitive and 83 promotive reactions were extracted (Supplementary File S4). Since one metabolic reaction may participate in multiple IRs, some metabolic reactions may have different effects on ecdysone synthesis in different IRs. The exact effect of these reactions on ecdysone needs to be determined by further calculations. The reactions affecting ecdysone synthesis include a complete pathway for ecdysone synthesis, which produces ecdysone from cholesterol, T00112 (transport for cholesterol) --> R11007 --> R08132 --> R08133 --> R08134 --> R08135 --> T00113 (transport for ecdysone). The presence of this pathway demonstrates the accuracy of using MPIN to calculate the elements that influence ecdysone synthesis.

The pathway and subsystem of the 97 reactions influencing ecdysone synthesis were further analyzed. Table 6 shows the distribution of these reactions in the KEGG pathways. The pathways enriched of competitive and promotive reactions are generally consistent, suggesting that the ecdysone metabolism is closely related to the pathways listed in Table 6 and some reactions in these pathways promote ecdysone synthesis, while some others have competitive effects. The pathway with the greatest impact on ecdysone synthesis is arginine and proline metabolism, followed by steroid biosynthesis and insect hormone biosynthesis pathways.

#### 3.3.2. Analysis of Proteins Affecting Ecdysone Synthesis

**Figure 5:**
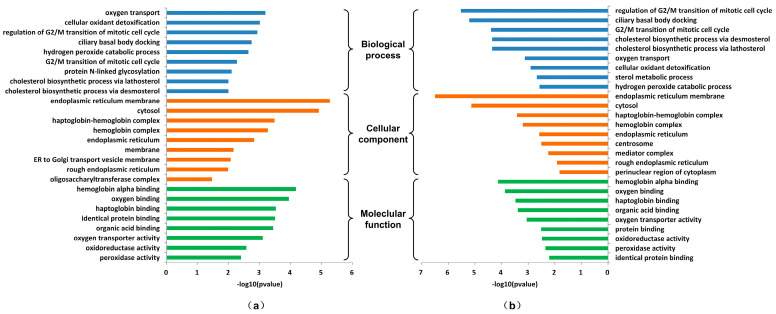
The GO enrichment analysis of the promotive and inhibitor proteins. (**a**) The GO enrichment analysis of the promotive proteins; (**b**) the GO enrichment analysis of the inhibitor proteins.

The IRs that affect ecdysone synthesis in the MPIN contain 113 proteins, including 96 promotive proteins and 94 inhibitor proteins. The GO enrichment analysis of these proteins was performed with the DAVID database. Figure 5 shows the results of the GO enrichment analysis. In addition to the metabolic processes such as the cholesterol biosynthetic process, hydrogen peroxide catabolic process, and oxygen transport, the promotive proteins are mainly concentrated in the transition of the mitotic cell cycle and protein N-linked glycosylation. The former is a regulatory process, and the latter is a protein modification process. The inhibitor proteins are also associated with the regulation of the cholesterol biosynthetic process and the transition of the mitotic cell cycle. In addition, many proteins are enriched in the sterol metabolic process to compete with cholesterol synthesis. The simultaneous prediction of complex processes such as metabolism, regulation, and protein modification can only be achieved by integrated networks, demonstrating the superiority of MPIN over single networks.

#### 3.3.3. Analysis of Key Reactions and Proteins Affecting Ecdysone Synthesis

**Figure 6:**
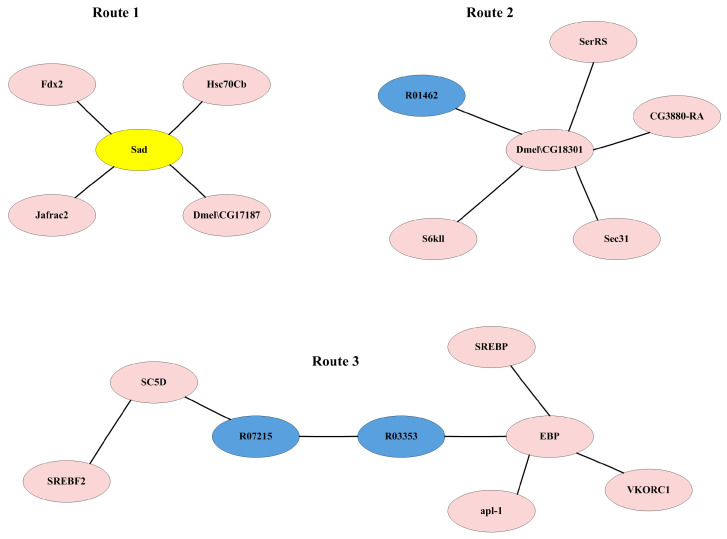
Key IRs significantly affecting ecdysone synthesis. Pink nodes indicate proteins, blue nodes indicate reactions, and yellow nodes indicate proteins in the ecdysone synthesis pathway.

There are 236 IRs affecting ecdysone synthesis obtained in Section 3.2.2. In order to determine the reactions having a significant impact on the ecdysone synthesis, the flux of each IR was fixed at its maximum and minimum values, respectively, and the maximum synthesis rates of ecdysone were simulated and compared to determine the impact of each individual IR on ecdysone synthesis, as described in Method Section 2.5. As a result, most IRs have a quite small influence on the ecdysone synthesis. There are 16 IRs that have a significant impact on the synthesis rate of ecdysone with changes of over 3% (Supplementary File S5). These IRs constitute three synthesis routes including three metabolic reactions from the GSMN and sixteen proteins from the PIN (Figure 6).

The first route is the classic ecdysone synthesis pathway which is affected by the protein Shadow (Sad). Sad is a cytochrome P450. It is located in the mitochondria and catalyzes the last step of ecdysone synthesis by transforming 2-deoxyecdysone to ecdysone. The second route contains the Dmel\CG18301 protein, which acts as an ester bond hydrolase to catalyze R01462, which releases cholesterol by breaking down cholesterol esters and thereby influences the synthesis of ecdysone.

In the third route, Apl-1 interacts with EBP to influence metabolic reactions. Apl-1 is a necessary protein required for larval transformation and morphogenesis processes, especially in the molting stage [31]. As a sterol isomerase, EBP mainly catalyzes the conversion of sterols. It catalyzes R03353 for the formation of lathosterol. SREBF2 interacts with SC5D as a sterol regulatory element binding protein, and SC5D catalyzes R07215. The existence of R03353 and R07215 form a route to generate cholesterol from cholestenol, i.e., R03353 (C03845 --> C01189) --> R07215 (C01189 --> C01164) --> R01451 (C01164 --> C00187), which subsequently affects the synthesis of ecdysone. Cholestenol can be synthesized through the steroid biosynthesis pathway.

R03353/R07215 and R01462 changed the synthesis rate of ecdysone by 12.5% and 50%, respectively. Therefore, it is possible that *E. sinensis* has the ability to synthesize cholesterol from other substances instead of absorbing it from feeds directly. In addition, compared with the number of IRs influencing the synthesis of ecdysone, the number of key IRs is quite small, which indicates that the synthesis and regulation of ecdysone synthesis is a complex process influenced by the combined effect of multiple reactions and proteins. The low number of key reactions may be due to the presence of alternative synthesis routes.

## 4. Conclusions

Integration of large-scale biological networks is an important step towards whole-cell network reconstruction. Multi-network integration has always been a challenge in biological network research. In this work, the GSMN and PIN of *E. sinensis* were integrated to reconstruct the first MPIN for higher animals, which included 1827 metabolic reactions, 228 transfer reactions, and 8225 proteins. The MPIN was used for the simulation of ecdysone synthesis. Through the flux variation analysis, 236 IRs were found to have a relationship with the synthesis of ecdysone, in which 16 IRs had a significant impact. These IRs constitute three synthesis routes including three metabolic reactions from the GSMN and sixteen proteins from the PIN. It is hypothesized that a route from lathosterol may exist in *E. sinensis*. The key proteins in the synthesis routes provide important regulation information of the ecdysone synthesis. The analysis based on the MPIN shows the complex influence elements influencing the synthesis of ecdysone, including metabolism, regulation, and protein modification. Furthermore, the results of this work provide a new progression for the integration and simulation of complex large-scale biological networks.

## Figures and Tables

**Table 1:** The scale of GSMN as a reaction graph.

Item	Count
Reaction	1793
Reversible-metabolic-reaction	405
Irreversible-metabolic-reaction	1388
Exchange reaction	89
Transport reaction	89
R-R interactions	4636
Reversible R-R interaction	756
Irreversible R-R interaction	3562
Reversible T-R interaction	73
Irreversible T-R interaction	245
Metabolites	1658

**Table 2:** Scale of preliminary reconstructed MPIN of *E. sinensis*.

Item	Count
Nodes	10,301
Protein	8225
Metabolic reaction	1793
Transport reaction	89
Exchange reaction	89
IRs	153,305
P-P IR	147,656
R-R IR	4318
T-T IR	89
T-E IR	89
T-R IR	318
P-R IR	835

**Table 3:** The added nodes and interactions after the addition of biomass equation.

Item	Count
New-Nodes	2
B00001[c]	1
B00001[e]	1
New-IRs	148
R-B IR	128
T-B IR	20
T-T IR	1
T-E IR	1

**Table 4:** The scale of the MPIN of *E. sinensis*.

Item	Number
Nodes	10,267
Protein	8225
Metabolic reaction	1816
Transport	112
Exchange	112
Biomass	2
IR	153,611
P-P IR	147,656
R-R IR	4364
T-T IR	113
T-E IR	113
T-R IR	391
P-R IR	835
R-B IR	128
T-B IR	20

**Table 5:** The scale of MPIN after adding the ecdysone synthesis pathway.

Item	Number
Nodes	10,282
Protein	8225
Metabolic reaction	1827
Transport reaction	114
Exchange reaction	114
Biomass reaction	2
IR	153,647
P-P IR	147,656
R-R IR	4376
T-T IR	115
T-E IR	115
T-R IR	396
P-R IR	841
R-B IR	128
T-B IR	20

**Table 6:** Pathway distribution of the competitive and promotive metabolic reactions.

Subsystem	Pathway	Competitive Reaction	Promotive Reaction
Glucose metabolism	Glycolysis/Gluconeogenesis	1	3
	Citrate cycle (TCA cycle)	1	2
	Pentose phosphate pathway	4	3
	Pentose and glucuronate interconversions	3	1
	Galactose metabolism	1	1
	Inositol phosphate metabolism	2	2
	Pyruvate metabolism	2	1
	Propanoate metabolism	1	1
	GPI-anchor biosynthesis	0	1
Amino acid metabolism	Arginine biosynthesis	1	1
	Alanine, aspartate and glutamate metabolism	1	1
	Glycine, serine and threonine metabolism	1	1
	Valine, leucine and isoleucine degradation	1	3
	Arginine and proline metabolism	11	17
	Tyrosine metabolism	2	1
	β-Alanine metabolism	1	2
	D-Amino acid metabolism	3	2
Lipid metabolism	Steroid biosynthesis	7	5
	Glycerolipid metabolism	3	6
	Glycerophospholipid metabolism	2	4
	Sphingolipid metabolism	1	1
Cofactor, vitamin metabolism	Ubiquinone and other terpenoid-quinone biosynthesis	1	2
	Folate biosynthesis	1	3
Nucleic acid metabolism	Pyrimidine metabolism	1	4
Metabolism of Terpenoids and Ketones	Insect hormone biosynthesis	2	9

## Data Availability

Data are contained within the article.

## Supplementary Materials

The following supporting information can be downloaded at https://www.mdpi.com/article/10.3390/genes15040410/s1, Supplementary File S1: Construction of biomass equation; Supplementary File S2: Nodes and IRs in MPIN; Supplementary File S3: IRs affecting the synthesis of ecdysone; Supplementary File S4: Reactions and proteins affecting the synthesis of ecdysone; Supplementary File S5: IRs having a significant impact on the synthesis rate of ecdysone.

## Abbreviations

Genome-scale metabolic network (GSMN); protein–protein interaction network (PIN); metabolic–protein interaction integrated network (MPIN); integrated reaction (IR); reaction–reaction integrated reaction (R-R IR); protein–reaction integrated reaction (P-R IR); protein–protein integrated reaction (P-P IR); transport–reaction integrated reaction (T-R IR); transport–transport integrated reaction (T-T IR); transport–exchange integrated reaction (T-E IR); reaction–biomass integrated reaction (R-B IR); transport–biomass integrated reaction (T-B IR).
