# Supplementary material for: Reconstruction of Metabolic–Protein Interaction Integrated Network of Eriocheir sinensis and Analysis of Ecdysone Synthesis
Source: Genes (Basel). 2024 Mar 26;15(4):410. doi: 10.3390/genes15040410 (PMC11049885; doi:10.3390/genes15040410)
Supplement: Supplementary file 1 [file genes-15-00410-s001.zip › Supplementary file 1.pdf]

Supplementary file 1 Construction of biomass equation

substance	content	unit	coefficient	compound ID
H2O	54.2	g/100g	65.6889	C00001
Glucose	0.777	g/100g	0.0942	C00267
Fructose	0.268	g/100g	0.0325	C02336
ASP	0.6786	g/100g	0.1113	C00049
THR	0.3499	g/100g	0.0641	C00188
SER	0.27775	g/100g	0.0577	C00065
GLU	0.8508	g/100g	0.1263	C00025
GLY	0.38925	g/100g	0.1132	C00037
ALA	0.45235	g/100g	0.1109	C00041
VAL	0.38635	g/100g	0.072	C00183
MET	0.16695	g/100g	0.0244	C00073
ILE	0.29345	g/100g	0.0488	C00407
LEU	0.54125	g/100g	0.0901	C00123
TYR	0.42925	g/100g	0.0517	C00082
PHE	0.3457	g/100g	0.0457	C00079
LYS	0.4455	g/100g	0.0665	C00047
HIS	0.1846	g/100g	0.026	C00135
ARG	0.44565	g/100g	0.0559	C00062
PRO	0.30265	g/100g	0.0574	C00148
TRP	0.08	g/100g	0.0085	C00078
CYS	0.07	g/100g	0.012	C00097
ASN	0.4745	g/100g	0.0784	C00152
GLN	0.4745	g/100g	0.0709	C00064
Lauric acid	0.0427	g/100g	0.0047	C02679
Tridecylic acid	0.0221	g/100g	0.0023	M00001
Myristic acid	0.920	g/100g	0.088	C06424
Myristoleic acid	0.0990	g/100g	0.0095	C08322
Pentadecylic acid	0.261	g/100g	0.0235	C16537
Palmitic acid	7.55	g/100g	0.6429	C00249
Palmitoleic acid	3.45	g/100g	0.2961	C08362
Margaric acid	0.146	g/100g	0.0118	M00002
cis-10- Heptadecenoic acid	0.217	g/100g	0.0177	M00003
Stearic acid	1.05	g/100g	0.0806	C01530
Elaidic acid	0.0654	g/100g	0.0051	C01712
Oleic acid	7.87	g/100g	0.6083	C00712
Linoleic acid	4.40	g/100g	0.3426	C01595
Arachidic acid	0.119	g/100g	0.0083	C06425
γ-Linolenic acid	0.0265	g/100g	0.0021	C06426
cis-11-Eicosenoic acid	0.686	g/100g	0.0482	C16526
α-Linolenic acid	0.502	g/100g	0.0394	C06427
HeneiCosanoic acid	0.0512	g/100g	0.0034	M00004

all cis-11,14 -Eicosadienoic acid	0.223	g/100g	0.0158	C16525
Behenic acid	0.0581	g/100g	0.0037	C08281
Dihomo-γ-linolenoic acid	0.0421	g/100g	0.003	C03242
Erucic acid	0.0818	g/100g	0.0053	C08316
all cis-11,14,17-Eicosatrienoic acid	0.107	g/100g	0.0076	C16522
Arachidonic acid	0.412	g/100g	0.0295	C00219
Tricosanoic acid	0.0376	g/100g	0.0023	M00005
all cis-13,16-Docosadienoic acid	0.0177	g/100g	0.0011	C16533
Lignoceric acid	0.0523	g/100g	0.0031	C08320
all cis-5,8,11,14,17-Eicosapentaenoic acid	1.36	g/100g	0.0982	C06428
Nervonic acid（Selacholeic)	0.107	g/100g	0.0064	C08323
all cis-4,7,10,13,16,19-Docosahexaenoic acid	3.37	g/100g	0.224	C06429
P	174	mg/100g	0.1227	C06262
Ca	1.56	g/kg	0.085	C00076
Cu	10.5	mg/kg	0.0004	C00070
Fe	74.3	mg/kg	0.0029	C14819
K	1.46	g/kg	0.0815	C00238
Mg	307	mg/kg	0.0276	C00305
Mn	2.02	mg/kg	0.0001	C00034
Na	2.26	g/kg	0.2146	C01330
Zn	16.9	mg/kg	0.0006	C00038
Se	0.41	mg/kg	0.00001	C01529
CMP	0.33	mg/100g	0.00002	C00055
UMP	0.96	mg/100g	0.00006	C00105
IMP	2.02	mg/100g	0.00013	C00130
GMP	0.5	mg/100g	0.00003	C00144
AMP	0.5	mg/100g	0.00003	C00020
ATP			29.8303	C00002
ADP			29.8303	C00008
hydron			29.8303	C00080
phosphate			29.8303	C00009

Biomass reaction: B00001

65.6889 C00001 + 0.0942 C00267 + 0.0325 C02336 + 0.1113 C00049 + 0.0641

C00188 + 0.0577 C00065 + 0.1263 C00025 + 0.1132 C00037 + 0.1109 C00041 + 0.072 C00183 + 0.0244 C00073 + 0.0488 C00407 + 0.0901 C00123 + 0.0517 C00082 + 0.0457 C00079 + 0.0665 C00047 + 0.026 C00135 + 0.0559 C00062 + 0.0574 C00148 + 0.0085 C00078 + 0.012 C00097 + 0.0784 C00152 + 0.0709 C00064 + 0.0047 C02679 + 0.0023 M00001 + 0.088 C06424 + 0.0095 C08322 + 0.0235 C16537 + 0.6429 C00249 + 0.2961 C08362 + 0.0118 M00002 + 0.0177 M00003 + 0.0806 C01530 + 0.0051 C01712 + 0.6083 C00712 + 0.3426 C01595 + 0.0083 C06425 + 0.0021 C06426 + 0.0482 C16526 + 0.0394 C06427 + 0.0034 M00004 + 0.0158 C16525 + 0.0037 C08281 + 0.003 C03242 + 0.0053 C08316 + 0.0076 C16522 + 0.0295 C00219 + 0.0023 M00005 + 0.0011 C16533 + 0.0031 C08320 + 0.0982 C06428 + 0.0064 C08323 + 0.224 C06429 + 0.1227 C06262 + 0.085 C00076 + 0.0004 C00070 + 0.0029 C14819 + 0.0815 C00238 + 0.0276 C00305 + 0.0001 C00034 + 0.2146 C01330 + 0.0006 C00038 + 0.00001 C01529 + 0.00002 C00055 + 0.00006 C00105 + 0.00013 C00130 + 0.00003 C00144 + 0.00003 C00020 + 29.8303 C00002 --> 29.8303 C00008 + 29.8303 C00009 + 29.8303 C00080 + Biomass
